# Effects of the Japanese Encephalitis Virus Genotype V-Derived Sub-Viral Particles on the Immunogenicity of the Vaccine Characterized by a Novel Virus-Like Particle-Based Assay

**DOI:** 10.3390/vaccines7030081

**Published:** 2019-08-04

**Authors:** Sarah Honjo, Michiaki Masuda, Tomohiro Ishikawa

**Affiliations:** Department of Microbiology, Dokkyo Medical University School of Medicine, 880 Kita-kobayashi, Mibu-machi, Shimotsuga-gun, Tochigi 321-0293, Japan

**Keywords:** Japanese encephalitis virus, genotype V, vaccine, virus-like particle, sub-viral particle, immunogenicity

## Abstract

Japanese encephalitis virus (JEV) is classified into five genotypes labelled I through V. Although the genotype V (GV) JEV was originally found and had apparently been limited in Malaysia for more than 50 years, its emergence in Korea and China has recently been reported. Therefore, the GV JEV might be spreading over new geographical regions as a cause of potential public health problems. However, it is unknown whether the currently available JEV vaccines are effective against the emerging GV strains. To investigate this issue, a novel virus-like particle-based neutralizing assay was developed in this study. By using this assay, the inactivated JEV vaccine used in Japan and the recombinant sub-viral particles (SVPs) bearing the E protein of the GV Muar strain were characterized for the immunogenicity against the GV JEV. Although the inactivated vaccine alone failed to elicit a detectable level of neutralizing antibodies against the GV JEV, the vaccine added with the Muar-derived SVPs induced relatively high titers of neutralizing antibodies, associated with the efficient Th1 immune responses, against the GV JEV. The results indicate that addition of the GV JEV-derived antigens may be useful for developing the vaccine that is universally effective against JEV including the emerging GV strains.

## 1. Introduction

The Japanese encephalitis virus (JEV), which belongs to the genus *flavivirus* of the family of *Flaviviridae*, is a major cause of viral encephalitis in Asia. Although most JEV infection is asymptomatic [1], symptomatic cases range from mild manifestations, such as diarrhea, headache, and vomiting, to severe forms including seizure, polio-like flaccid paralysis, meningitis, or encephalitis [2]. The typical fatality rate of the symptomatic cases is considered to be 20–30% [3]. Moreover, 30–50% of the cases with severe symptoms result in psychoneurological sequelae.

JEV is maintained in the mosquito—bird/swine cycle in nature and is transmitted to humans by the vector mosquito, *Culex tritaeniorhynchus* [4]. JEV possesses an approximately 11 kb-long positive-stranded RNA as a genome, which encodes three structural proteins, capsid (C), precursor membrane (prM), and the envelope (E), as well as seven non-structural (NS) proteins, NS1, NS2A, NS2B, NS3, NS4A, NS4B, and NS5 [5]. Based on the nucleotide sequences of the structural protein genes, JEV is classified into five distinct genotypes, which is labelled I through V [6]. Until the 1990s, the genotype III (GIII) JEV had been widely spread and most frequently isolated in the southern, southeastern, and eastern Asia. However, it was replaced by genotype I (GI) in many countries including Japan, South Korea, Vietnam, and Thailand [7]. The Muar strain of JEV was originally isolated from an encephalitis patient in Malaysia in 1952 [8] and later classified as genotype V (GV) [9]. Although the GV JEV had long been found only in Malaysia, isolation in China of a new GV strain designated as XZ0934 from the mosquito was reported in 2009 [10]. In Korea, the RNA molecules corresponding to the GV JEV genome were detected in mosquitoes in 2010 [11] and in 2012 [12]. Although the GV JEV has not extensively been characterized, recent studies revealed its potent virulence and neuro-invasiveness in mice [13,14]. These previous studies suggest that the GV JEV, which may already have colonized in Eastern Asia, could cause public health problems.

To prevent JEV infection, several vaccines are currently licensed in the world, including the cell-culture derived inactivated vaccine, the live-attenuated vaccine, and the chimeric vaccine [15]. All of these currently available vaccines contain antigens derived from the GIII JEV. These vaccines have been shown to elicit high titers of neutralizing antibodies against JEV of GI through genotype IV (GIV) [16]. On the other hand, it has been reported that the JEV vaccine currently used in Japan elicits lower titers of neutralizing antibodies against the Muar strain, which is the prototype GV JEV, than against GI and GIII strains [14].

In this study, immunogenicity of the JEV vaccine against the GV strains emerging in Eastern Asia was examined. For this purpose, a novel neutralization assay was developed by using the virus-like particles (VLPs) carrying a reporter gene-expressing sub-genomic replicon. By using the assay, effects of the sub-viral particle (SVP) antigens derived from the GV JEV on the immunogenicity of the vaccine against the emerging GV strains were also examined. The results indicated that, while the immunogenicity of the JEV vaccine currently used in Japan against the emerging GV JEV was relatively poor, addition of the GV-derived SVP antigens led to abundant production of the neutralizing antibodies, as well as the Th1 immune responses, against the emerging GV JEV. Therefore, addition of the GV JEV-derived antigens may be useful for the development of the vaccine that is universally effective against JEV including the emerging GV strains.

## 2. Materials and Methods

### 2.1. Cells and Viruses

Vero and BHK cells were grown at 37 °C with 5% CO_2_ in Eagle’s minimum essential media (MEM) and Dulbecco’s modified MEM, respectively, which was supplemented with 10% fetal bovine serum (FBS), 10 mM nonessential amino acid (NEAA, Thermo Fisher Scientific, Waltham, MA, USA), and penicillin (1000 unit/mL)-streptomycin (100 µg/mL) (PS). Mosquito-derived C6/36 cells were grown at 30 °C with 5% CO_2_ in MEM supplement with 10% FBS, 10 mM NEAA, and PS. Nakayama and Muar strains of JEV were prepared in C6/36 cells and Vero cells, respectively, as described previously [17]. After inoculation with the virus or VLPs, cells were maintained in MEM supplemented with 1% FBS, 10 mM NEAA, and 10 mM 2- [4- (2-Hydroxyethyl) -1-piperazinyl] ethanesulfonic acid (HEPES) (MEM-1%FBS). To collect SVPs and VLPs, cells were maintained after electroporation in 10% VP-SFM (Thermo Fisher Scientific)-90% MEM supplemented with 10 mM HEPES and 10mM NEAA (VPSFM-MEM).

### 2.2. Preparation of VLPs and SVPs

**Figure 1:**
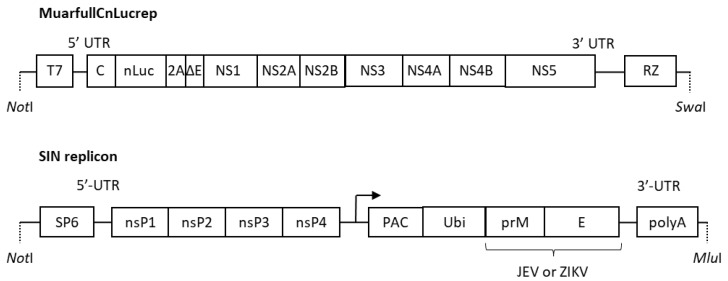
Structure of MuarfullCnLucrep and the Sindbis virus replicon (SIN replicon). MuarfullCnLucrep and the SIN replicon were constructed into a pACYC177-based plasmid and a pToto1101-based plasmid, respectively. The backbone structures of these plasmids were omitted in this figure. NS—non-structural protein of Japanese encephalitis virus. 2A—foot-and-mouse disease virus derived 2A protein. RZ; hepatitis delta virus ribozyme. nsP—non-structural protein of sindbis virus. PAC—puromycin N-acetyl-transferase. Ubi—ubiquitin. Arrow indicates secondary promotor of the sindbis virus genome.

Cloned DNA of the replicon, MuarLucrep, derived from the JEV Muar strain [17] was modified by substituting the firefly luciferase gene and the partially deleted C gene with the deep sea shrimp-derived smaller luciferase (NanoLuc) [18] of pNLVF1-N (Promega, Madison, WI, USA) and the full-length C gene to construct MuarfullCnLucrep (Figure 1). Plasmid DNA of MuarfullCnLucrep was linearized with *Swa*I digestion and then subjected to in vitro transcription by using a MEGAscript T7 Kit (Thermo Fisher Scientific, Waltham, MA, USA).

The DNA clone of the Sindbis virus sub-genomic replicon (SIN replicon) was constructed by inserting the Sindbis virus nonstructural protein genes under the SP6 promoter. In the downstream of the secondary promoter for the sub-genomic viral RNA, the fragment of the fusion protein consisting of puromycin N-acetyl-transferase, ubiquitin, as well as the C-terminal 20 amino acids of the C protein, and the prM-E protein of JEV and Zika virus (ZKV) strains, was inserted (Figure 1). The JEV strains from which the prM-E gene was derived were Nakayama and Muar strains, and the emerging GV strains with amino acid substitution (R84K, V186I, P171L or A343V; Table 1) in the E protein, which was compared to Muar strain. The PRVABC59 strain was used for ZKV. Each replicon plasmid was linearized by *Mlu*I digestion and then used as a template for in vitro transcription with a MEGAscript SP6 kit (Thermo Fisher Scientific, Waltham, MA, USA).

For preparation of VLPs, C6/36 cells were introduced by electroporation [17] with a mixture of the RNA samples derived from MuarfullCnLucrep (8 μg) and one of the SIN replicons (8 μg). Then, the cells were maintained in VPSFM-MEM, and culture fluids were collected every 24 h for 4 days and pooled. The VLPs in the pooled samples were concentrated by using Amicon Ultra-15, 100K (Merck, Darmstadt, Germany), according to the manufacturer’s instruction.

The SVPs were prepared similarly except that BHK cells were introduced with in vitro-transcribed SIN replicon RNA alone (16 μg). An aliquot of the SVP samples was subjected to polyacrylamide gel electrophoresis and silver staining, and the amount of the proteins in SVPs was estimated by comparing the band densities with those of known amounts of proteins.

### 2.3. Immunization of Mice

Four-week-old ICR mice (5–6 mice/immunogen) were immunized twice via the intraperitoneal route with a two-week interval. As immunogens, Vero cell-derived inactivated JE Beijing-1 vaccine (JEVAX, BIKEN, Osaka, Japan) approved and currently used in Japan (1/10 of the normal adult dose in 200 μL of saline), Muar-derived SVP (Mu-SVP, 800 ng), or a mixture of JEVAX (1/10 dose) and Mu-SVP (400 ng) were used. Saline alone was used as the control. Three weeks after the secondary immunization, mice were euthanized, and whole blood was collected from each mouse. Collected blood samples were incubated at 37 °C for 30 min and then centrifuged at 3000 rpm for 10 min to obtain serum samples, which were stored at −30 °C until further use. All of the animal experiments were conducted in accordance with the guidelines of the Laboratory Animal Research Center, Dokkyo Medical University, by using the approved protocol (no. 1028).

### 2.4. Neutralization Test

Neutralization tests by using the infectious JEV were conducted as described previously [19] with minor modifications. Pooled mouse sera were heat inactivated at 56 °C for 30 min, serially diluted by two-fold and incubated with JEV at 37 °C for 1 h. Then, the mixture was inoculated onto Vero cells seeded in a 96-well plate. After absorption for 2 h, cells were overlaid with 1% methylcellulose in MEM supplemented with 1% FBS. Forty-eight hours later, the cells were fixed with the mixture of 50 *v/v*% acetone- 50 *v/v*% methanol, and the foci of virus-infected cells were visualized by immunostaining with an anti-JEV E monoclonal antibody 10B4 [19]. The neutralizing antibody titer was expressed as the highest serum dilution yielding 70% reduction in the number of foci.

The neutralizing tests by using VLPs were performed similarly except that, after absorption, inoculum was removed, and the cells were grown in MEM-1% FBS for 48 h, rinsed with phosphate buffer saline (PBS), and used for the luciferase assay. The luciferase activities of the cell lysates were measured by using the Nano-Glo Luciferase Assay System (Promega, Madison, WI, USA), according to the manufacturer’s instructions. Briefly, 25 µl of the cell lysate was mixed with 25 µl of substrate, and the mixture was incubated for 3 min. Then, the luminescence was measured for 10 s by Lumat LB9501 (Berthold Technologies, Bad Wilbad, Germany). The neutralization titer was expressed as the highest serum dilution yielding 70% reduction in the relative light unit.

### 2.5. Enzyme-Linked ImmunoSorbent Assay (ELISA)

A 96-well EIA plate (Corning Inc., Corning, NY, USA) was sensitized with concentrated SVPs (approximately 2 µg protein/well) in carbonate buffer (pH 9.6) at 4 °C overnight. After rinsing with PBS-0.05% Tween-20, each well was blocked with PBS containing 0.05% Tween-20 and 1% bovine serum albumin for 20 min and then added with individual sera diluted at 1:100. Following incubation at room temperature for 2 h, the plate was incubated with HRP-labeled anti-mouse IgG (GE Healthcare, Chicago, IL, USA). Prior to incubation with the substrate from an ELISA POD Substrate TMB Kit (Nacalai Tesque, Kyoto, Japan), the plate was rinsed with PBS-0.05% Tween-20 and then with water. The reaction was stopped by adding 1N H2SO4. The optical density (OD) was measured on the iMark Microplate Reader (Bio Rad, Hercules, CA, USA).

For Th1/Th2 profiling, a 96-well plate was sensitized as described above, and pooled mouse serum samples serially diluted by two-fold were added to each well and incubated at room temperature for 2 h. Then, the plate was reacted with the HRP-labeled anti-mouse IgG1 or IgG2a antibody (Southern Biotechnology Associates, Birmingham, UK) and then the substrate was added after a rinse with PBS-0.05% Tween-20 and water. The optical density (OD) was measured as described above. The IgG1 and IgG2a antibody titers were expressed as the highest serum dilution exhibiting OD greater than the average plus three times the standard deviation obtained for the serum sample of the saline-inoculated mouse.

## 3. Results

### 3.1. Preparation of the Luciferase Gene-Transducing VLP Bearing the E Protein of JEV and ZKV

**Figure 2:**
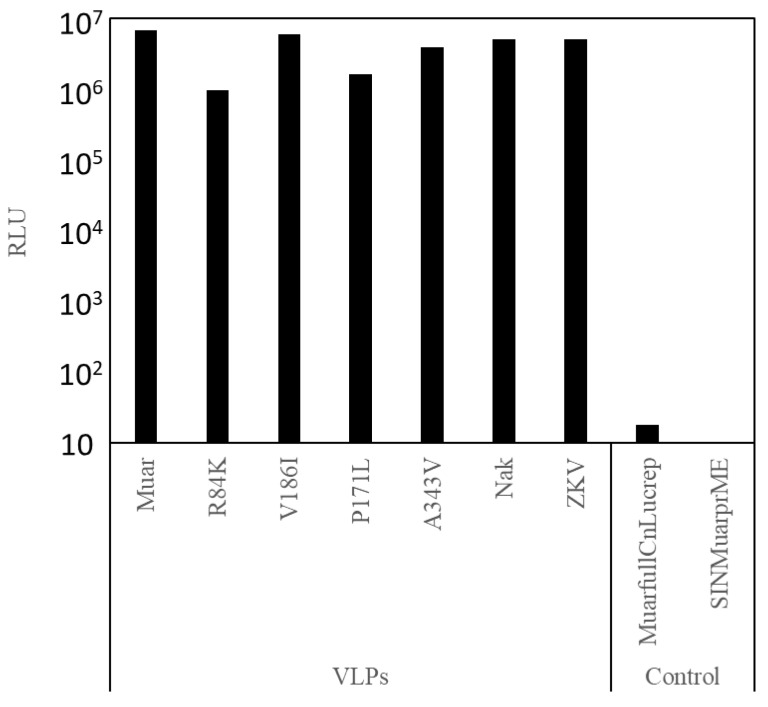
Production of nLuc-expressing virus like particles (VLPs). The nLuc-expressing VLP obtained from C6/36 cells electroporated with MuarfullCnLucrep RNA and sindbis virus replicon expressing prM-E genes were inoculated to Vero cells. Cells were lysed at 48 h post inoculation and then nano-luciferase activities were measured.

The luciferase-expressing replicon (MuarfullCnLucrep) RNA was introduced into C6/36 cells with the SIN replicon RNA bearing the prME gene derived from the GIII Nakayama strain or various GV strains (Muar, R84K, V186I, P171L or A343V) of JEV, or ZKV. Then, the pooled culture fluids corresponding to respective SIN replicons were inoculated onto Vero cells, and luciferase activities of the cell lysates were measured at 48 h post-inoculation. As shown in Figure 2, high levels of luciferase activity were detected for all of the seven SIN replicons, but not the control MuarfullCnLucrep or SIN replicon alone. The data indicate the successful transduction of luciferase gene mediated by the VLPs bearing the E protein expressed by the SIN replicon.

### 3.2. Evaluation of the VLP for Application in the Neutralization Test

Mice were immunized with 1/10 dose of JEVAX, and the sera obtained from these mice were tested for the neutralization titers by using infectious JEV (Nakayama strain) and the VLP bearing the E protein derived from the Nakayama strain. As expected, JEVAX was able to elicit antibody production (1:20) against the Nakayama strain, which belongs to GIII. Similarly, neutralization antibodies (1:10) were detected against the VLP bearing the E protein of the Nakayama strain. When the VLP bearing the ZKV E protein was used, the neutralization titer was shown to be <1:10. Therefore, the VLP bearing the JEV E protein appeared to serve as a useful tool for measuring the virus-specific neutralization antibody titer even though the sensitivity might be slightly lower than the infectious virus.

### 3.3. Immunogenicity of JEVAX and JEV Muar Strain-Derived SVP Against JEV GV

Mice were immunized with JEVAX (1/10 dose), the Muar strain-derived SVP (Mu-SVP), or the mixture of these two, and the sera obtained from these mice were examined for the neutralization titers by the VLP-based assay described above. Whereas mice immunized with JEVAX alone did not elicit a detectable level of antibody production against any of the JEV GV strains examined, immunization with Mu-SVP alone developed neutralizing antibodies (1:10) against most of the JEV GV strains examined except R84K (Table 2). Unexpectedly, the Mu-SVP-induced neutralization titer (1:20) against Nakayama strain, which belongs to GIII, was higher (Table 2). Immunization with the mixture of JEVAX with Mu-SVP led to considerably higher neutralization titers against JEV GV (1:40), as well as GIII (1:80) (Table 2). No detectable levels of neutralization antibody production against ZKV were induced by any of these immunization protocols.

**Figure 3:**
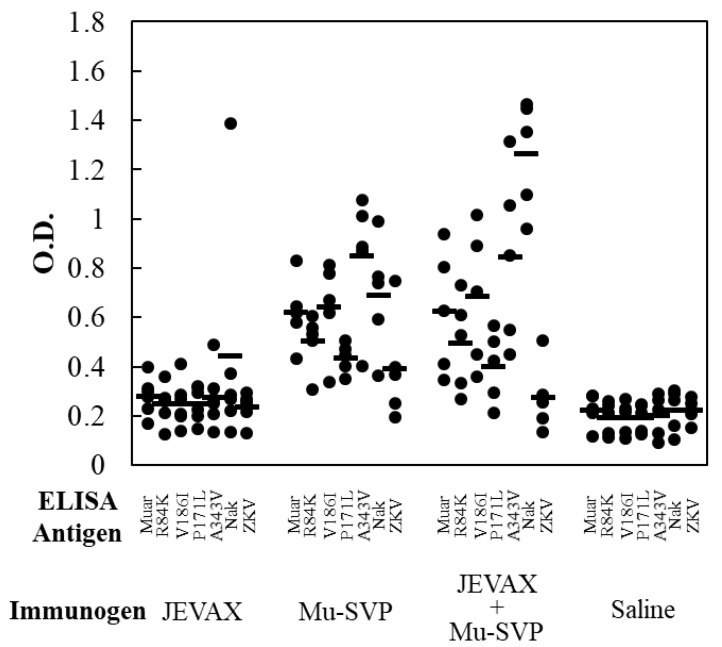
ELISA antibody levels induced by JEVAX and/or Muar-derived sub-viral particle (Mu-SVP). Antibody levels of individual mouse sera were examined on the antigen-sensitized plates. SVPs (Muar, R84K, V186I, P171L, A343V, Nak, and ZKV) were used as antigens. The individual sera were analyzed and bars represent the average of each group.

The levels of antibody production were also measured by ELISA (Figure 3). The results appeared to be correlated with those of the neutralization test. The highest antibody levels were observed in the sera from mice immunized with the mixture of JEVAX and Mu-SVP, which was followed by Mu-SVP alone and JEVAX alone (Figure 3). Intriguingly, the antibody level in the sera from Mu-SVP-immunized mice against the Nakayama strain tended to be higher than against most of the GV strains except A343V. In most cases, no significant levels of antibody production against ZKV were observed with any of the immunization protocols (Figure 3).

### 3.4. Th1/Th2 Immune Profile Induced by JEVAX and Mu-SVP

**Figure 4:**
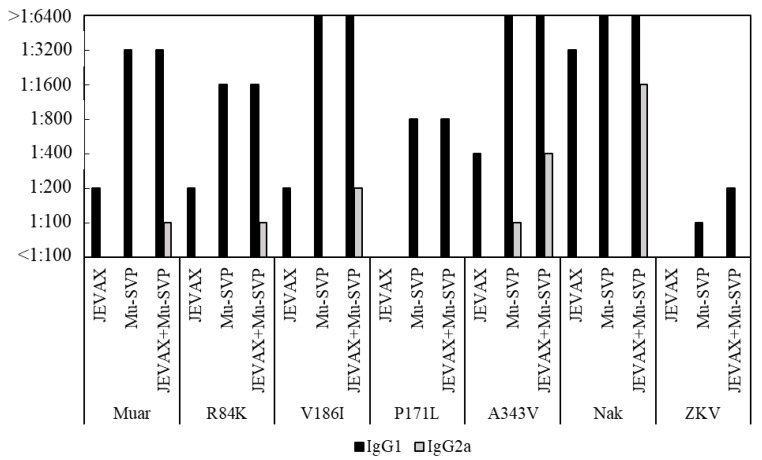
The Th1/Th2 immune profile induced by JEVAX and/or Muar-derived subviral particle (Mu-SVP). The serial two-fold diluted pooled mouse sera (start from 1:100) were examined on the antigen-sensitized plates. SVPs (Muar, R84K, V186I, P171L, A343V, Nak, and ZKV) were used as antigens. Antibody titers of IgG1 (Th2) and IgG2a (Th1) of each group were determined as the highest serum dilution exhibiting optical density (O.D.) greater than average O.D. plus three times the standard deviation obtained by saline inoculated mouse sera.

Th1 cells are known to promote cellular immune responses while Th2 cells promote humoral immune responses. In mice, the degrees of Th1 and Th2 immune responses are represented by the levels of IgG2a and IgG1 antibodies, respectively [20]. The isotype-specific ELISA revealed that production of IgG1, but not IgG2a, was elicited by immunization with JEVAX against the GV strains except P171L against which neither IgG1 nor IgG2a were detected (Figure 4). Similarly, the IgG1 titers were markedly higher than the IgG2a titers when Mu-SVP alone or the mixture of JEVAX and Mu-SVP was used as immunogen. The results indicate that the Th2-dominant immune responses were generally elicited by all of the immunogens tested. However, it should be noted that Th1 immune responses represented by production of IgG2a against the Nakayama strain and most of the GV strains examined were also observed when the mixture of JEVAX and Mu-SVP was used (Figure 4).

## 4. Discussion

For prevention of the JEV infection, effective vaccines have been developed and utilized in the endemic areas. For evaluating the immunogenicity of the vaccines, the titers of the neutralizing antibodies in the immunized sera have usually been measured by the plaque reduction assay. However, this assay needs an infectious virus and generally takes at least a week to obtain the results. Recently, the neutralizing assays by using the VLPs, which carry the reporter gene-expressing sub-genomic replicon, have been developed for some flaviviruses [19,21,22,23,24]. These VLP-based assays for which infectious virus is unnecessary are thought to be safe. In addition, the reporter VLP assay can be performed in a few days and could be applicable for high throughput tests. In this study, a novel VLP-based neutralization assay was developed by modifying the previous system [19] and shown to be useful for measuring the anti-JEV antibody titers.

All of the currently approved JEV vaccines, which contain antigens derived from the GIII strains, are known to be effective against the GI through GIV viruses [16]. It has previously been reported that the inactivated JEV vaccine, which contains the antigens derived from the GIII Beijing-1 strain, is poorly immunogenic against the Muar strain, which is the prototype GV JEV isolated in Malaysia decades ago [14]. Our present study confirmed the previous results by using the VLP-based neutralization assay and demonstrated that the GIII-based vaccine was not effective against the emerging GV strains reported from Eastern Asia. Our results are consistent with the previous study showing that the live attenuated vaccine derived from the GIII SA14-14-2 strain induced only low titers of neutralizing antibodies against the GV XZ0934 strain recently isolated in China [25]. Relatively poor immune responses by JEVAX were observed in this study. The neutralizing antibody titers were determined in this study by a 70% reduction method, which is often used for evaluating a novel vaccine, whereas the 50% reduction method is quality assurance of existing vaccine evaluations. In addition, sensitivities of the VLP-based neutralization test might be slightly lower than the infectious virus-based test, as mentioned above. These might be a reason for the low neutralization titers by JEVAX. Regarding the ELISA assay, structural differences between immunogen (JEVAX) and antigens used in ELISA evaluation (SVPs), and differences in cells used to prepare JEVAX (Vero cells) and SVPs (BHK cells) might be reasons for lower ELISA values.

The envelope of the individual JEV particle is estimated to bear 180 molecules of E protein, which plays crucial roles in receptor binding and membrane fusion. The E protein is highly immunogenic, and, thus, most neutralizing antibodies against JEV react with the E protein [5]. The poor immunogenicity of the GIII-based vaccine against the GV viruses is most likely due to the lower amino acid identities of the E protein between GIII and GV. In fact, the amino acid sequence identities of the E protein between the GIII and GV strains are about 90%, whereas those between GIII and GI, GII, or GIV are 94% or higher [8]. Neutralizing monoclonal antibodies (mAbs) derived from the GIII JEV-infected mice have been shown to recognize specific amino acids in the E protein. For example, the 503 mAb recognized Q52, I126, K136, and S275 [26], and neutralization by the E3.3 mAb was shown to require S331 and D332 [27]. Among these critical residues, Q52 is substituted in all GV viruses except the Muar strain, and S331 is substituted to T in all GV viruses examined (Table 1). The amino acid at the positions 52 and 331 are located on the surface of the E protein, and their substitution might affect the accessibility and, thus, the neutralizing activity of the antibodies elicited by the GIII-based vaccine. The amino acid identities of the E protein between JEV and ZKV are only 54–55%. Reasonably, no significant levels of neutralizing antibodies were detected in mice immunized with the JEVAX or Mu-SVP.

As to flaviviruses, it is known that the cells expressing only prM-E gene can produce viral RNA-free SVPs, which bear E and M proteins in the envelope [28]. Our present study demonstrated that the Mu-SVP served as a potent immunogen for eliciting neutralizing antibodies against various strains of GV JEV except R84K. Unexpectedly, the neutralizing antibodies raised by Mu-SVP to Nak were higher than homologous GV viruses, even though the mechanism of this observation is unknown. For the West Nile virus, which also belongs to the genus flavivirus, it was reported that at least approximately 30 antibodies per single virion were required for neutralization [29]. Therefore, it is possible that the GV virus might require a larger number of antibodies to be neutralized compared to the GIII virus. Interestingly, a mixture of JEVAX and Mu-SVP exhibited the enhanced immunogenicity against not only the GV strains but also the GIII strain, which suggests the synergistic effects between the vaccine and the SVPs. For the protection from JEV, the neutralizing antibody titer at 1:10 has been accepted as immunological correlates [30]. Thus, the neutralizing antibodies induced particularly by the mixture of JEVAX and Mu-SVP were considered protective. Although the exact mechanism for the enhancement is unknown, it is possible that the SVPs may act as an adjuvant as well as antigens because the SVPs (about 30 nm in diameter) are smaller than the inactivated virus particles (50–70 nm) and could easily be drained into lymph nodes and recognized by the resident dendritic cells and macrophages [31]. These enhancements might result from a simple mixture of antigens derived from different genotypes. To investigate this possibility, a comparison of immunogenicity of Mu-SVP and a Muar-based inactivated vaccine will be valuable for further study.

In general, viral infection and immunization with live attenuated vaccines elicit the Th1-dominant immune responses [32,33], while immunization with inactivated or subunit vaccines usually induces the Th2-dominant immune responses [32]. Consistently, our data indicated that the Th2-dominant immune responses were elicited by JEVAX. Additionally, the Th2-dominant immune responses were induced when Mu-SVPs were injected alone, likely because the SVPs, unlike live attenuated vaccines, are replication-defective. Although IgG1 antibodies were elevated to GV viruses by JEVAX alone, no detectable neutralizing antibodies were observed. The IgG1 as well as IgG2a levels shown in Figure 4 contain total anti-JEV antibodies whether they have neutralizing activities or not, which is likely causing these differences. Intriguingly, a mixture of the vaccine and the SVPs were able to elicit notable levels of Th1 immune responses represented by the production of IgG2a. Although Th2 immune responses have been recognized as a major contributor for protection, Th1 immune responses were also reported to play supplementary roles in the protection of JEV [34]. Therefore, induction of both Th1 and Th2 immune responses by our vaccine is considered useful. Although the profile of the T cell responses are known to be affected by several factors, such as the route for vaccine inoculation and the adjuvant used with the immunogen [35,36,37], it is unknown how the Th1 immune responses were elicited when the two immunogens were mixed. Further studies are necessary to elucidate the mechanism.

Taken together, the new VLP-based neutralizing assay demonstrated that immunogenicity against the prototype and the emerging GV strains of the JEV vaccine currently used in Japan was relatively poor. However, the addition of Mu-SVP increased the vaccine’s immunogenicity against the GV viruses apparently in a synergistic manner. Additionally, the vaccine added with Mu-SVP was able to elicit the Th1 immune responses more effectively than either immunogen alone. Therefore, the addition of the vaccine with the GV-derived antigens may be useful for developing the new JEV vaccine universally effective against a wide range of genotypes including GV.

## Figures and Tables

**Table 1:** Amino acid substitutions among JEV GV strains.

Strain	Accession number *	Designation **	Amino Acid Position in E Protein										
			52	58	84	129	156	171	186	208	240	292	343
Muar	HM596272	Muar	Q	T	R	I	S	P	V	T	L	D	A***
K12AS1148	KJ420590	R84K	E	T	K	I	T	P	V	S	M	E	A
K12AS1151	KJ420591	R84K	E	T	K	I	T	P	V	S	M	E	A
10-1827	JN587258	R84K	E	T	K	I	T	P	V	S	M	E	A
XZ0934	JF915894	V186I	E	T	R	I	T	P	I	S	M	E	A
K12YJ1203	KJ420592	P171L	E	T	K	I	T	L	V	S	M	E	A
K12HC959	KJ420589	A343V	E	A	K	T	T	P	V	S	M	E	V
Beijing 1	AB920347	-	Q	S	K	T	S	P	V	S	M	D	A
Nakayama	AB920348	-	Q	S	K	T	S	P	V	S	M	D	A

* Gene bank accession number was shown. ** These designations were used hereinafter. ***Q: glutamine, E: glutamic acid, T: threonine, A: alanine, S: serine, R: arginine, K: lysine, I: isoleucine, P: proline, L: leucine, V: valine, M: methionine, D: aspartic acid.

**Table 2:** Immunogenicity of JEVAX and Mu-SVP.

Immunogen	Virus like particles Used in the Neutralization Test *						
	Muar	R84K	V186I	P171L	A343V	Nak	ZKV
1/10 dose of JEVAX	<1:10	<1:10	<1:10	<1:10	<1:10	1:10	<1:10
Mu-SVP** (800 ng)	1:10	<1:10	1:10	1:10	1:10	1:20	<1:10
1/10 dose of JEVAX and Mu-SVP (400 ng)	1:40	1:40	1:40	1:40	1:40	1:80	<1:10
Saline	<1:10	<1:10	<1:10	<1:10	<1:10	<1:10	<1:10

* Neut titers were expressed as the highest serum dilution yielding 70% reduction in a relative light unit. ** Muar-derived sub-viral particle.
